# Serologic Surveillance and Phylogenetic Analysis of SARS-CoV-2 Infection Among Hospital Health Care Workers

**DOI:** 10.1001/jamanetworkopen.2021.18554

**Published:** 2021-07-28

**Authors:** Jonne J. Sikkens, David T. P. Buis, Edgar J. G. Peters, Mireille Dekker, Michiel Schinkel, Tom D. Y. Reijnders, Alex. R. Schuurman, Justin de Brabander, A. H. Ayesha Lavell, Jaap J. Maas, Jelle Koopsen, Alvin X. Han, Colin A. Russell, Janke Schinkel, Marcel Jonges, Sébastien Matamoros, Suzanne Jurriaans, Rosa van Mansfeld, W. Joost Wiersinga, Yvo M. Smulders, Menno D. de Jong, Marije K. Bomers

**Affiliations:** 1 Department of Internal Medicine, Amsterdam Infection and Immunity Institute, Amsterdam University Medical Centers, Vrije Universiteit Amsterdam, Amsterdam, the Netherlands; 2 Section Infectious Diseases, Department of Internal Medicine, Amsterdam Infection and Immunity Institute, Amsterdam University Medical Centers, Vrije Universiteit Amsterdam, Amsterdam, the Netherlands; 3 Department of Medical Microbiology and Infection Prevention, Amsterdam Infection and Immunity Institute, Amsterdam University Medical Centers, Vrije Universiteit Amsterdam, Amsterdam, the Netherlands; 4 Center for Experimental Molecular Medicine, Amsterdam Infection and Immunity Institute, Amsterdam University Medical Centers, University of Amsterdam, Amsterdam, the Netherlands; 5 Department of Occupational Health and Safety, Amsterdam University Medical Centers, University of Amsterdam, Amsterdam, the Netherlands; 6 Department of Medical Microbiology and Infection Prevention, Amsterdam University Medical Centers, University of Amsterdam, Amsterdam, the Netherlands; 7 Division of Infectious Diseases, Department of Internal Medicine, Amsterdam Infection and Immunity Institute, Amsterdam University Medical Centers, University of Amsterdam, Amsterdam, the Netherlands

## Abstract

**Importance:**

It is unclear when, where, and by whom health care workers (HCWs) working in hospitals are infected with SARS-CoV-2.

**Objective:**

To determine how often and in what manner nosocomial SARS-CoV-2 infection occurs in HCW groups with varying exposure to patients with COVID-19.

**Design, Setting, and Participants:**

This cohort study comprised 4 weekly measurements of SARS-CoV-2–specific antibodies and collection of questionnaires from March 23 to June 25, 2020, combined with phylogenetic and epidemiologic transmission analyses at 2 university hospitals in the Netherlands. Included individuals were HCWs working in patient care for those with COVID-19, HCWs working in patient care for those without COVID-19, and HCWs not working in patient care. Data were analyzed from August through December 2020.

**Exposures:**

Varying work-related exposure to patients infected with SARS-CoV-2.

**Main Outcomes and Measures:**

The cumulative incidence of and time to SARS-CoV-2 infection, defined as the presence of SARS-CoV-2–specific antibodies in blood samples, were measured.

**Results:**

Among 801 HCWs, there were 439 HCWs working in patient care for those with COVID-19, 164 HCWs working in patient care for those without COVID-19, and 198 HCWs not working in patient care. There were 580 (72.4%) women, and the median (interquartile range) age was 36 (29-50) years. The incidence of SARS-CoV-2 was increased among HCWs working in patient care for those with COVID-19 (54 HCWs [13.2%; 95% CI, 9.9%-16.4%]) compared with HCWs working in patient care for those without COVID-19 (11 HCWs [6.7%; 95% CI, 2.8%-10.5%]; hazard ratio [HR], 2.25; 95% CI, 1.17-4.30) and HCWs not working in patient care (7 HCWs [3.6%; 95% CI, 0.9%-6.1%]; HR, 3.92; 95% CI, 1.79-8.62). Among HCWs caring for patients with COVID-19, SARS-CoV-2 cumulative incidence was increased among HCWs working on COVID-19 wards (32 of 134 HCWs [25.7%; 95% CI, 17.6%-33.1%]) compared with HCWs working on intensive care units (13 of 186 HCWs [7.1%; 95% CI, 3.3%-10.7%]; HR, 3.64; 95% CI, 1.91-6.94), and HCWs working in emergency departments (7 of 102 HCWs [8.0%; 95% CI, 2.5%-13.1%]; HR, 3.29; 95% CI, 1.52-7.14). Epidemiologic data combined with phylogenetic analyses on COVID-19 wards identified 3 potential HCW-to-HCW transmission clusters. No patient-to-HCW transmission clusters could be identified in transmission analyses.

**Conclusions and Relevance:**

This study found that HCWs working on COVID-19 wards were at increased risk for nosocomial SARS-CoV-2 infection with an important role for HCW-to-HCW transmission. These findings suggest that infection among HCWs deserves more consideration in infection prevention practice.

## Key Points

**Question:**

Which hospital health care workers are at increased risk for SARS-CoV-2 infection, and by whom are they infected?

**Findings:**

In this cohort study of 801 hospital health care workers (HCWs), the risk of getting infected with SARS-CoV-2 was nearly 4-fold higher among HCWs on COVID-19 wards compared with HCWs not in patient care. Combined phylogenetic and epidemiological analyses found no patient-to-HCW transmission but several occurrences of HCW-to-HCW transmission.

**Meaning:**

These findings suggest that transmission of SARS-CoV-2 between HCWs deserves more consideration in infection prevention practice.

## Teaser

This cohort study investigates prevalence and transmission routes of SARS-CoV-2 infection among hospital health care workers in the Netherlands using serologic surveillance and phylogenetic and epidemiological analyses.

## Introduction

In 2020, health care institutions worldwide were overwhelmed by large numbers of patients with COVID-19. Stringent infection prevention and control measures have been applied to prevent transmission from patients to health care workers (HCWs) and from HCWs to HCWs. Nonetheless, HCWs have become infected during provision of care for patients with COVID-19, and there is ongoing debate concerning transmission dynamics^1^ and which infection prevention and control measures are adequate.^2,3,4^ Delivering direct care to patients with COVID-19 has been associated with infection or COVID-19–related hospital admission among HCWs in some^5,6,7,8,9^ but not all studies.^10,11,12,13,14^ Most studies were cross-sectional and retrospective and lacked predefined control groups and detailed information on SARS-CoV-2 exposure, including use of personal protective equipment (PPE).

To quantify the incidence of SARS-CoV-2 infection among HCWs, identify potential risk factors associated with infection, and elucidate potential transmission routes, we performed the Serologic Surveillance of SARS-CoV-2 Infection in Health Care Workers (S3) study in 2 tertiary care medical centers in the Netherlands. Participants were working during the first wave of SARS-CoV-2-infections. Serial serologic measurements and epidemiological data were combined with phylogenetic analysis of viruses isolated from patients and HCWs to identify transmission clusters.

## Methods

### Study Design and Population

We conducted a prospective serologic surveillance cohort study among HCWs of the Amsterdam University Medical Centers in the Netherlands, which comprises 2 tertiary care hospitals. Measurements of SARS-CoV-2–specific antibodies were taken every 4 weeks over 18 weeks during the first COVID-19 wave (ie, March 23-June 25, 2020). The first patient with a confirmed COVID-19 diagnosis was admitted on March 9. Enrolment of HCWs took place from March 23 to April 7 except for HCWs in non–COVID-19 care, who were enrolled during the final measurement, in June 2020. Phlebotomies were combined with surveys, which included questions on personal and work-related SARS-CoV-2 exposure and symptoms. Recruitment of HCWs was done by leaflets distributed in relevant departments with potentially eligible HCWs and by intranet news items. Participants were invited for and reminded of follow-up measurements by email.

Potential participants were eligible for inclusion in 1 of 3 specific groups based on exposure to patients with COVID-19: (1) HCWs working as nurses or physicians with bedside contacts with patients with COVID-19 on designated regular-care COVID-19 wards, emergency departments (EDs), or intensive care units (ICUs); (2) HCWs working as nurses or physicians on wards designated for non–COVID-19 care; and (3) HCWs not working in patient care. The second group participated in only the final measurement.

This study was reviewed and approved by institutional review boards of both hospitals, and written informed consent was obtained from each participant. The study report follows the Strengthening the Reporting of Observational Studies in Epidemiology (STROBE) reporting guideline.

### Infection Prevention Practices

The tertiary care centers instituted identical infection prevention and control measures in accordance with European and national guidelines.^15,16^ Initially, all HCWs caring for patients suspected of having COVID-19 used PPE consisting of disposable nonsterile gloves, gowns, FFP2 masks (which are considered equivalent to N95 masks), and reusable goggles.^17^ From March 16 onward, national guidelines on PPE were adjusted in accordance with recommendations at the time.^15,16^ Type IIR surgical masks were used during non–aerosol-generating care, and FFP2 masks were used during intensive care and high-risk, aerosol-generating procedures (ie, high-flow nasal oxygen therapy, noninvasive ventilation, intubation, bronchoscopy, and nebulized medication). No PPE was recommended outside direct care of patients with COVID-19, but social distancing measures were implemented through the hospitals (eg, keeping 1.5 m of distance between individuals, conducting no meetings with >30 people or with external visitors, and closing sitting areas in restaurants). Additional details regarding infection practices are provided in eMethods in the Supplement.

### Procedures

We collected survey data using Castor Electronic Data Capture version 2020.1 (Castor).^18^ A survey example is provided in eMethods in the Supplement. At each measurement, participants reported results of any preceding SARS-CoV-2 nucleic acid amplification test (NAAT) of nasopharyngeal swabs, which were performed as part of routine hospital testing of symptomatic HCWs. Measurement in serum of SARS-CoV-2–specific antibodies was done using the Wantai SARS-CoV-2 pan-immunoglobulin anti-S1-receptor-binding domain test according to the manufacturer’s instructions (Beijing Wantai Biological Pharmacy enzyme-linked immunosorbent assay [ELISA], Bioscience chemiluminescence immunoassay, and Zhuhai Livzon ELISA).^19^ Indeterminate results were classified as negative.

### Outcomes

The primary outcomes were cumulative incidence of and time to SARS-CoV-2 infection during the study period. Infection with SARS-CoV-2 was defined as presence of SARS-CoV-2–specific antibodies above the threshold set by the manufacturer. The date of SARS-CoV-2 infection was defined as the sampling date of a first positive NAAT result or, in its absence, the midpoint between the last seronegative and the first seropositive sample. All participants were assumed to be seronegative on February 27, which was 4 weeks before the first measurement and the day of the first diagnosis of COVID-19 in the Netherlands.

Outcomes were compared among the 3 study groups. Subgroup analysis included comparisons between hospital unit types (ie, COVID-19 ward, ICU, and ED) and profession (ie, nurse and physician). Secondary outcomes included results of phylogenetic analyses and infection rates by self-reported exposure to patients with COVID-19, number of household contacts with COVID-19, and presence of COVID-19-associated symptoms

### Viral Sequencing and Phylogenetic Analyses

To identify possible transmission clusters, virus sequencing was performed from routinely stored nasopharyngeal swabs of 26 HCWs who were infected (including study participants and others) and 39 patients with COVID-19. These HCWs and patients were selected from COVID-19 wards with the highest incidence of infection among HCWs from which the largest number of temporally associated patient samples were also available. Included HCWs worked on COVID-19 wards from March 15 to May 15; included patients had been admitted to corresponding wards from March 13 to April 19. Complete viral genomes were sequenced using the Ion AmpliSeq SARS-CoV-2 Research Panel, Ion Chef, and Ion Torrent S5 platforms (all Thermo Fisher Scientific). Consensus full-length SARS-CoV-2 genomes (ie, >29 000 nucleotide bases long with minimum depth of coverage for each site of 100 bases) were generated by removing reads ends with Phred quality scores of less than 20 using Trimmomatic version 0.39 and mapping raw reads against the WIV04 reference genome (GenBank reference MN996528.1) using Bowtie 2 version 2.4.1.^20,21,22^

We used Mafft version 7.427 (Research Institute for Microbial Diseases) to align SARS-CoV-2 sequences from HCWs and patients, together with 300 randomly selected, contemporaneous SAR-CoV-2 virus genomes from the Netherlands (see eAppendix in the Supplement for Global Initiative on Sharing Avian Influenza Data [GISAID] accession numbers).^23^ We inferred a maximum likelihood tree with IQ-Tree version 2.0.6 (Minh et al^24^) using the Hasegawa-Kishino-Yano (HKY) + proportion of invariable sites (I) + gamma-distributed rate variation among sites (G) model. We applied Phydelity version 1 (Han et al) to the maximum likelihood tree to infer putative transmission clusters.^25^

We used Bayesian Evolutionary Analysis Sampling Trees (BEAST) version 1.10.4 (BEAST Developers) to reconstruct a bayesian time-scaled phylogenetic tree for the same set of sequences using the HKY + I + G model with a strict molecular clock, an exponential growth prior, and an informative clock prior based on recent estimates of SARS-CoV-2 substitution rate (Γ distribution prior with a mean of 0.8 × 10^−3^ substitutions/site/y and an SD of 5 × 10^−4^).^26,27^ We performed and combined 2 chains of 100 million steps. Convergence was reached for all parameters (effective sample size > 700).

### Statistical Analysis

We used Kaplan-Meier estimates with log-rank test and univariable and multivariable Cox regression analyses to compare SARS-CoV-2 infection over time between study groups. The proportional hazard assumption did not hold because of fluctuating incidence of COVID-19 during the study period, evidenced by Schoenfeld tests resulting in *P* < .05. The reported hazard ratios [HRs] should therefore be interpreted as mean relative hazards for the entire study period instead of a relative hazard at each individual time point. Multivariable models contained all other covariates used in the univariable models; these covariates were selected based on clinical relevance. Analysis was based on individuals with complete data on covariates included in the regression models. Hypothesis testing was 2-sided, and results were considered statistically significant when 95% CIs did not cross 1. R statistical software version 4.0.3 (R Project for Statistical Computing) was used for all other analyses. Data were analyzed from August through December 2020.

## Results

### Participants

Among 801 HCWs, there were 439 HCWs in the COVID-19 care group, 164 HCWs in the non–COVID-19 care group, and 198 HCWs in the no patient care group. We excluded 1 additional participant during the first measurement because this individual did not meet inclusion criteria. Median (interquartile range [IQR]) age was 36 (29-50) years, and there were 580 (72.4%) women. The HCWs providing COVID-19 and non–COVID-19 care were younger than HCWs not working in patient care (median [IQR] age, 34 [29-44]) years and 33 [27-49], respectively, vs 49 [40-57] years) (Table 1). Median [IQR] follow-up duration was 120 [92-120] days with a maximum of 120 days. For measurements 2 through 4, survey completion rates were higher than the rate of HCWs complying with blood sampling, which was likely associated with measurements 2 through 4 not requiring physical presence. None of the participants with a SARS-CoV-2 infection reported being hospitalized during the study period (eTable 1 in the Supplement).

**Table 1.** General Characteristics of Participants

Characteristic, No. (%)^a^	COVID-19 care (n = 439)	Non–COVID-19 care (n = 164)	No patient care (n = 198)
Age, median (IQR)	34 (29-44)	33 (27-49)	49 (40-57)
Sex^b^			
Women	289 (70.3)	145 (88.4)	146 (76.4)
Men	122 (29.7)	19 (11.6)	45 (23.6)
Position			
Nurse	219 (49.9)	129 (78.7)	0
Resident	107 (24.4)	25 (15.2)	0
Specialist	86 (19.6)	10 (6.1)	0
Other patient care	27 (6.2)	0	0
Administration or policy	0	0	62 (31.3)
Scientist	0	0	43 (21.7)
Pharmacy	0	0	17 (8.6)
Other nonpatient care	0	0	76 (38.4)
Tertiary care center			
Amsterdam University Medical Centers, location Academic Medical Center	253 (57.6)	73 (44.5)	84 (42.4)
Amsterdam University Medical Centers, location Vrije Universiteit University Medical Center	186 (42.4)	91 (55.5)	114 (57.6)
Days/week spent in hospital, mean (range)	4.1 (1-5.5)	3.8 (2-5.5)	2.9 (1-5.5)
Serology test result			
Ever positive	54 (12.3)	11 (6.7)	7 (3.5)
PCR test result			
Ever positive	27 (6.2)	7 (4.3)	0
Always negative	165 (37.6)	58 (35.4)	20 (10.1)
Never tested	247 (56.3)	99 (60.4)	178 (89.9)
PPE training followed			
Electronic learning only	160 (36.6)	65 (39.6)	NA
Simulation only	13 (3)	3 (1.8)	NA
Both	253 (57.9)	92 (56.1)	NA
None	11 (2.5)	4 (2.4)	NA
Feasibility of social distancing			
Easy	2 (0.5)	11 (6.7)	42 (21.9)
Medium	27 (6.5)	11 (6.7)	56 (29.2)
Difficult	111 (26.6)	46 (28)	69 (35.9)
Virtually impossible	278 (66.5)	96 (58.5)	25 (13)
Worried about getting COVID-19			
Not at all	117 (33.9)	102 (62.2)	52 (29.7)
Somewhat	164 (47.5)	37 (22.6)	92 (52.6)
Medium	53 (15.4)	16 (9.8)	26 (14.9)
Very	11 (3.2)	9 (5.5)	5 (2.9)
If worried, most worried about			
Personal health	53 (23.3)	17 (27.4)	28 (22.8)
Infecting friends or family	156 (68.7)	40 (64.5)	90 (73.2)
Infecting patients	18 (7.9)	5 (8.1)	0
Infecting colleagues	0	0	5 (4.1)

Abbreviations: IQR, interquartile range; NA, not applicable; PCR, polymerase chain reaction; PPE, personal protective equipment.

^a^ Data are from a survey among health care workers.

^b^ Information on sex was missing for 35 participants because they did not participate in measurement 2, when this was asked as part of the survey.

### Primary Outcome

**Figure 1.**
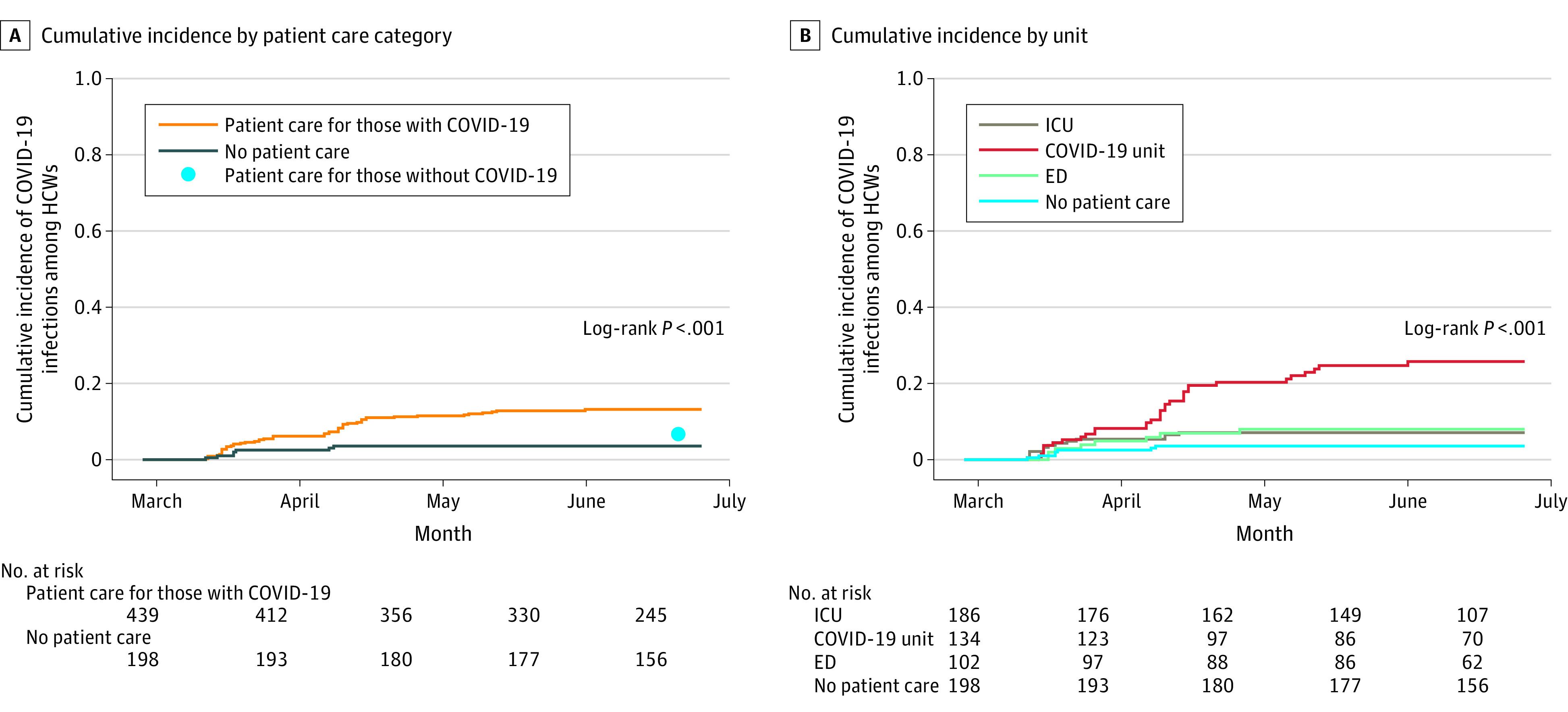
Cumulative Incidence of COVID-19 Among Health Care Workers (HCWs) ED indicates emergency department; ICU, intensive care unit.

Cumulative incidence of SARS-CoV-2 was increased among HCWs working in COVID-19 care (54 HCWs [13.2%; 95% CI, 9.9%-16.4%]) compared with HCWs in non–COVID-19 care (11 HCWs [6.7%; 95% CI, 2.8%-10.5%]; HR, 2.25; 95% CI, 1.17-4.30) and in HCWs not working in patient care (7 HCWs [3.6%; 95% CI, 0.9%-6.1%]; HR, 3.92; 95% CI, 1.79-8.62) (Figure 1A; Table 2). Among HCWs caring for patients with COVID-19, SARS-CoV-2 cumulative incidence was increased among HCWs working on COVID-19 wards (32 of 134 HCWs [25.7%; 95% CI, 17.6%-33.1%]) compared with HCWs working on ICUs (13 of 186 HCWs [7.1%; 95% CI, 3.3%-10.7%]; HR, 3.64; 95% CI, 1.91-6.94) and HCWs working in EDs (7 of 102 HCWs [8.0%; 95% CI, 2.5%-13.1%]; HR, 3.29; 95% CI, 1.52-7.14) (Figure 1B; Table 2). The number of COVID-19 admissions to study hospitals and regional COVID-19 incidence are shown in eFigure 1 in the Supplement. Results were similar for individual study sites (eFigure 2A and B and eFigure 3A and B in the Supplement) and when including only NAAT or only serology results in the analysis (eFigure 2C and D and eFigure 3C and D in the Supplement). Main results were similar after adjustment in multivariable Cox regression.

**Table 2.** Univariable and Multivariable Cox Regression Analysis of Factors Associated With SARS-CoV-2 Infection

Factor^a^	SARS-CoV-2 incidence, No./total No. (%; 95% CI)^b^	HR (95% CI)	Adjusted HR (95% CI)^c^
**Overall study population**			
HCW work environment			
No patient care	7/198 (3.6; 0.9-6.1)	1 [Reference]	1 [Reference]
Non–COVID-19 care only	11/164 (6.7; 2.8-10.5)	1.75 (0.68-4.51)	1.59 (0.58-4.37)
COVID-19 care	54/439 (13.2; 9.9-16.4)	3.92 (1.79-8.62)^d^	3.08 (1.23-7.66)^d^
Contact with coworker with COVID-19			
No	30/455 (7.0; 4.5-9.4)	1 [Reference]	1 [Reference]
Yes	40/319 (13.5; 9.5-17.3)	2.02 (1.26-3.24)	1.71 (0.99-2.97)
Contact with community member with COVID-19			
No	52/693 (8.2; 6.0-10.3)	1 [Reference]	1 [Reference]
Yes	20/108 (20.2; 11.8-27.9)	2.60 (1.55-4.35)	2.03 (1.14-3.62)
Days per week spent in hospital	NA	1.07 (0.84-1.35)	1.71 (0.99-2.97)
Age	NA	0.98 (0.96-0.997)	0.99 (0.97-1.01)
**Within care of patients with COVID-19**			
Hospital unit type			
ICU	13/186 (7.1; 3.3-10.7)	1 [Reference]	1 [Reference]
COVID-19 unit	32/134 (25.7; 17.6-33.1)	3.64 (1.91-6.94)^e^	3.71 (1.66-8.29)^e^
ED	7/102 (8.0; 2.5-13.1)	1.11 (0.46-2.67)	1.29 (0.46-3.61)
Combination	2/17 (11.9; 0-25.8)	2.03 (0.46-9.03)	1.21 (0.12-11.77)
Position			
Specialist	5/86 (6.4; 0.7-11.8)	1 [Reference]	1 [Reference]
Resident	14/107 (14.7; 7.5-21.3)	2.70 (0.98-7.42)	1.69 (0.50-5.67)
Nurse	35/246 (14.9; 10.2-19.3)	2.58 (1.01-6.59)	1.62 (0.57-4.60)
Self-reported COVID-19 exposure			
Low	13/88 (15.3; 7.3-22.7)	1 [Reference]	1 [Reference]
Medium	16/165 (11.2; 6.0-16.1)	0.65 (0.30-1.43)	0.50 (0.21-1.21)
High	13/73 (18.2; 8.7-26.6)	1.12 (0.52-2.42)	1.12 (0.43-2.90)
Very high	12/113 (10.8; 4.8-16.4)	0.66 (0.32-1.36)	0.77 (0.28-2.11)
Feasibility of social distancing			
Easy	0/2 (0; 0-0)	NA	NA
Medium	7/27 (26.7; 7.5-41.8)	1 [Reference]	1 [Reference]
Difficult	19/111 (18.5; 10.5-25.8)	0.60 (0.25-1.42)	0.42 (0.14-1.28)
Virtually impossible	26/278 (9.9; 6.3-13.4)	0.32 (0.14-0.73)	0.31 (0.11-0.90)
PPE always correctly used			
No	5/54 (9.4; 1.2-16.9)	1 [Reference]	1 [Reference]
Yes	49/383 (13.8; 10.2-17.3)	1.43 (0.57-3.59)	1.19 (0.41-3.45)
Contact with coworker with COVID-19			
No	17/186 (9.3; 5.0-13.5)	1 [Reference]	1 [Reference]
Yes	35/232 (16.0; 11.1-20.7)	1.71 (0.96-3.04)	2.36 (1.11-5.03)
Contact with community member with COVID-19			
No	39/360 (11.8; 8.2-15.1)	1 [Reference]	1 Reference]
Yes	15/79 (19.7; 10.2-28.2)	1.80 (0.996-3.26)	1.46 (0.70-3.05)
Days per week spent in hospital	NA	0.70 (0.52-0.94)	0.66 (0.47-0.92)
Age, y	NA	0.97 (0.95-1.00)	1.00 (0.97-1.03)

Abbreviations: ED, emergency department; HCW, health care worker; HR, hazard ratio; ICU, intensive care unit; NA, not applicable; PPE, personal protective equipment.

^a^ Data are from a survey among health care workers.

^b^ Percentages with CIs were calculated using the Kaplan-Meier method.

^c^ Adjusted HRs are for models containing all variables for the overall study population and for HCWs providing care to patients with COVID-19, respectively.

^d^ Compared with care of patients without COVID-19 only: HR, 2.25; 95% CI, 1.17-4.30; adjusted HR, 1.93; 95% CI, 0.98-3.81.

^e^ Compared with ED: HR, 3.29; 95% CI, 1.52-7.14; adjusted HR, 2.9X; 95% CI, 1.2X-7.1X.

Contact with an individual from the community (including the household) with COVID-19 (HR, 2.60; 95% CI, 1.55-4.35) and contact with a coworker with COVID-19 (HR, 2.02; 95% CI, 1.26-3.24) were associated with increased risk of COVID-19 infection (Table 2). Among 437 HCWs providing COVID-19 care, 426 HCWs (97.4%) followed training on the use of PPE. Self-reported adherence to PPE guidelines was mixed (131 of 138 HCWs on ICUs [94.9%], 133 of 149 HCWs on COVID-19 wards [89.3%], and 95 of 119 HCWs on EDs [79.8%]) but was not associated with SARS-CoV-2 incidence.

Among HCWs working in COVID-19 care, cumulative incidence among physicians was 19 individuals (11.0%; 95% CI, 6.3%-15.5%); specialists had decreased cumulative incidence compared with residents (5 individuals [6.4%; 95% CI, 0.7%-11.8%] vs 14 individuals [14.7; 95% CI, 10.2%-19.3%]; HR, 2.70; 95% CI, 0.98-7.42) and nurses (35 individuals [14.9%; 95% CI, 10.2%-19.3%]; HR, 2.58; 95% CI, 1.01 to 6.59).

Incidence of SARS-CoV-2 among HCWs was increased on 1 regular COVID-19-ward (ward 2) compared with other COVID-19 wards (eFigure 4 in the Supplement). This ward was similar to the other wards with regard to HCW deployment and architectural structure but had an increased proportion of patients with preexisting pulmonary disease and use of high-flow nasal oxygen therapy. To assess the contribution of this ward to overall results, the primary outcome was reanalyzed when excluding this ward, and we found a SARS-CoV-2 incidence among HCWs on COVID-19 units of 22 of 118 HCWs (19.7%; 95% CI, 12.0%-26.8%) (compared with HCWs on ICUs: HR, 2.78; 95% CI, 1.40-5.52) (eTable 2 in the Supplement).

### Secondary Outcomes

Among 72 participants with seroconversion, 33 participants (45.8%) also tested positive by NAAT during routine screening of symptomatic HCWs. Because of the restrictive access to SARS-CoV-2 testing at that time, all of these individuals were HCWs in direct patient care. There was 1 participant without documented seroconversion who tested positive by NAAT, which occurred prior to the fourth measurement. However, the subsequent blood sample was mislabeled and therefore not analyzed.

Among 72 HCWs with SARS-CoV-2 infection, 61 HCWs (84.7%) reported at least 1 symptom suggestive of COVID-19 (ie, cough, headache, sore throat, fever, dyspnea, chest pain, anosmia, cold, diarrhea) compared with 630 of 729 participants (86.4%) without infection. After adjustment for other symptoms, anosmia was associated with increased risk of infection: 39 of 72 participants who were seropositive (70.8%; 95% CI, 53.4%-81.7%) compared with 14 of 729 participants who were negative (4.5%; 95% CI, 3.0%-6.1%) (adjusted HR, 2.95; 95%, CI 13.71-45.41).

### Phylogenetic Analyses

**Figure 2.**
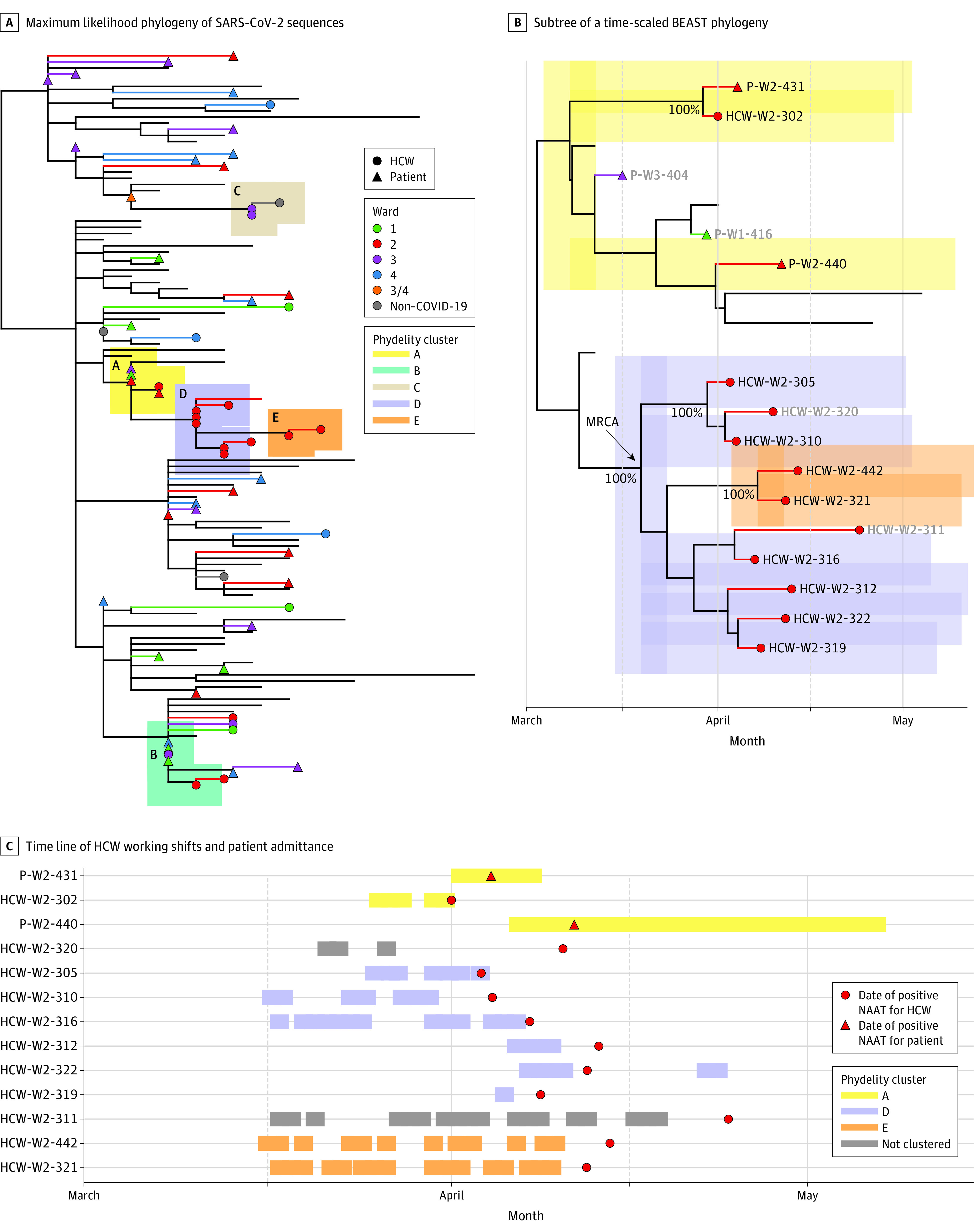
SARS-CoV-2 Sequence Phylogeny BEAST indicates Bayesian Evolutionary Analysis Sampling Trees; HCW, health care worker; MRCA, most recent common ancestor; and NAAT, nucleic acid amplification test.

In the maximum likelihood phylogeny, 32 of 39 sequences from patients admitted to a COVID-19 ward (Figure 2A) and 12 of 26 samples from HCWs were dispersed across the tree among 300 contemporaneous viruses from the Netherlands suggesting unrelated infections. Phydelity identified 5 putative transmission clusters containing the remaining 21 sequences (7 patients, 14 HCWs) (Figure 2A). Clusters A and B comprised patients clustering with each other or with HCWs. The 3 other transmission clusters (ie, C, D, and E) contained only HCWs.

Patient-to-patient and HCW-to-patient transmissions were unlikely because patients admitted to COVID-19 wards had NAAT-confirmed SARS-CoV-2 infection or were highly suspected of SARS-CoV-2 infection based on symptoms or radiological findings at time of admission. This is further evidenced by the lack of clear epidemiological links between patients in clusters A and B. There was additionally no evidence of patient-to-HCW transmission based on our phylogenetic analysis, and there was no overlap between the patient admission dates and HCW working shifts in clusters A and B (Figure 2C; eFigure 5 in the Supplement).

In 3 clusters containing only HCWs, there was a high degree of overlap in working shifts, suggesting epidemiological linkage (Figure 2C; eFigure 5 in the Supplement). In 2 clusters (ie, D and E), only sequences obtained from HCWs working in ward 2 were included. The time-scaled phylogeny (Figure 2B) suggests a single introduction for these HCWs working in ward 2 at approximately mid-March (median date, March 19, 2020; 95% highest posterior density interval: March 11-March 30, 2020; 100% posterior support).

## Discussion

In this cohort study, we prospectively followed a large cohort of HCWs during the first wave of the COVID-19 pandemic with the aim of comparing cumulative SARS-CoV-2 incidence between groups of HCWs with varying exposure to patients with COVID-19. We found a consistently increased risk of SARS-CoV-2 infection among HCWs caring for patients with COVID-19 compared with HCWs working in non–COVID-19 care and HCWs not working in patient care. In subgroup analysis, we found that the overall risk was largely associated with a substantially increased risk among HCWs on regular-care COVID-19 wards; infection rates among HCWs working in ICUs and EDs were similar to those among HCWs working in non–COVID-19 care. Our phylogenetic analysis combined with epidemiologic data identified transmission clusters comprising only HCWs, consistent with HCW-to-HCW transmission on COVID-19 wards, while no evidence of patient-to-HCW transmission was found.

Seroprevalence of SARS-CoV-2 among HCWs not working in patient care was similar to that of healthy blood donors in the Dutch general population at the time.^28^ The increased incidence among HCWs working in patient care of any kind suggests that working in patient care is associated with increased infection risk. Incidence of infection was highest among HCWs working in COVID-19 care, which may suggest that patient-to-HCW transmission was associated with the excess incidence in this group. However, we did not find an association between self-reported number of contacts who had COVID-19 and infection or between self-reported use of PPE and infection, which would have been expected if patient-to-HCW transmission was the dominant transmission pattern. Additionally, on 1 of 6 COVID-19 wards, multiple HCWs were infected before the first patient with COVID-19 was admitted. In the phylogenetic analyses, we also found no evidence for patient-to-HCW transmission, although this cannot be completely ruled out, given that nasopharyngeal samples were not available for all relevant patients or HCWs.

In phylogenetic analyses, we found evidence for HCW-to-HCW transmission on COVID-19 units. The hypothesis that HCW-to-HCW transmission played an important role was further supported by the increased incidence among HCWs who reported contact with a colleague who was SARS-CoV-2 positive. More than half of HCWs who were seropositive in our study did not report a positive NAAT result, suggesting that a significant proportion of infections among HCWs remained unrecognized. This suggests that HCWs likely have been working while unaware of their SARS-CoV-2 infections, hence presenting a risk of transmission. The number of HCWs present on COVID-19 wards was increased compared with other regular-care wards owing to the nature of care and because mobility of HCWs working in COVID-19 care through the hospital was discouraged. Personnel break rooms on COVID-19 wards were therefore more crowded than usual and more crowded than on non–COVID-19 care wards because of downscaling of regular care. While universal masking was not yet recommended during this period, it is arguable whether this would have made a difference in transmission in break rooms (or other places where HCWs would take breaks, eat, or drink) because masks cannot be worn while eating or drinking. The ICUs differed with regard to facilitating social distancing by using additional break rooms with clearly demarcated spaces between seats.

Preventing SARS-CoV-2 infection among HCWs is important to maintain the health of the individual HCW, to halt the ongoing pandemic, and to maintain a functioning health care system. Understandably, much attention has been focused on preventing patient-to-HCW transmission. Our results show that working in hospital patient care leaves HCWs at risk of infection through HCW-to-HCW transmission, which has received less attention and may deserve more consideration. We recommend in the current situation of high SARS-CoV2 incidence using optimal measures to facilitate social distancing on the work floor. These could include reducing the number of people per room by spreading out break times, increasing the size or number of break rooms, enabling online conferencing, recommending universal use of face masks, and investing in structural auditing and training by infection prevention and control personnel.

### Limitations

Our study has several limitations. First, despite the prospective cohort design, selection bias cannot be completely ruled out; for example, HCWs staying at home ill were not able to enroll if the absence happened during the first measurement, which may have resulted in underestimating of incidence. Second, not all nasopharyngeal samples from patients or HCWs collected for SARS-CoV-2 NAAT were available for viral sequencing analyses because they were not stored or the admitted patients were diagnosed elsewhere. Therefore, there may be missing clusters or missing links in the transmission clusters that were inferred. Third, no systematic data on compliance to infection prevention measures were collected, limiting more precise conclusions. Fourth, infection incidence was substantially increased on 1 COVID-19 ward, which also contributed most transmission clusters. This ward was the only non-ICU ward to use high-flow nasal oxygen therapy, which may have been associated with increased rates of patient-to-HCW transmission. However, we found no evidence for this in the transmission analysis so although a causative role cannot be completely excluded, it is unlikely to have played a major role. Importantly, when excluding this ward from the analysis, the proportion of HCWs who were seroconverted on regular COVID-19 wards remained more than 2-fold that found for ICUs. Fifth, although specificity of the Wantai serologic assay is reportedly high (99.3%), sensitivity is lower (85.2%, >15 days after symptom onset), so some false-negative results may have occurred.^19^ However, our repeated measurement design and the availability of NAAT results may have decreased the potential occurrence of such misclassification.

## Conclusions

These findings suggest that HCWs working on COVID-19 wards are at increased risk for nosocomial SARS-CoV-2 infection. Our results further suggest an important role for HCW-to-HCW transmission.

## Notes

Supplement.
eMethods.
**eAppendix.** Viral Genome Data Acknowledgement **eFigure 1.** COVID-19 Hospital and Intensive Care Admissions and Regional COVID-19 Incidence **eFigure 2.** Cumulative Incidence of SARS-CoV-2 Infection Among Health Care Workers With by Exposure to Patients With COVID-19 **eFigure 3.** Cumulative Incidence of SARS-CoV-2 Infection Among Health Care Workers With by Hospital Unit Type **eFigure 4.** Cumulative Incidence of SARS-CoV-2 Infection Among Health Care Workers per Specific COVID-19 Regular Ward **eFigure 5.** Working Shifts and Admittance Dates of Individuals Identified in Each Transmission Cluster **eTable 1.** Participant Characteristics by Measurement Moment **eTable 2.** Univariable and Multivariable Cox Regression Analysis Factors Associated With SARS-CoV-2 Infection After Exclusion of Ward 2
eReferences
